# Optimizing an Adaptive Neuro-Fuzzy Inference System for Spatial Prediction of Landslide Susceptibility Using Four State-of-the-art Metaheuristic Techniques

**DOI:** 10.3390/s20061723

**Published:** 2020-03-19

**Authors:** Mohammad Mehrabi, Biswajeet Pradhan, Hossein Moayedi, Abdullah Alamri

**Affiliations:** 1 Department of Civil Engineering, Kermanshah University of Technology, 6715685420 Kermanshah, Iran; mhmd.mehrabi@yahoo.com; 2 The Centre for Advanced Modelling and Geospatial Information Systems (CAMGIS), Faculty of Engineering and Information Technology, University of Technology, Sydney, NSW 2007, Australia; 3 Department of Energy and Mineral Resources Engineering, Sejong University, Choongmu-gwan, 209 Neungdong-ro Gwangjin-gu, Seoul 05006, Korea; 4 Informetrics Research Group, Ton Duc Thang University, Ho Chi Minh City, Vietnam; hossein.moayedi@tdtu.edu.vn; 5 Faculty of Civil Engineering, Ton Duc Thang University, Ho Chi Minh City, Vietnam; 6 Department of Geology & Geophysics, College of Science, King Saud Univ., P.O. Box 2455, Riyadh 11451, Saudi Arabia; amsamri@ksu.edu.sa

**Keywords:** landslide susceptibility, GIS, remote sensing, ANFIS, metaheuristic optimization

## Abstract

Four state-of-the-art metaheuristic algorithms including the genetic algorithm (GA), particle swarm optimization (PSO), differential evolutionary (DE), and ant colony optimization (ACO) are applied to an adaptive neuro-fuzzy inference system (ANFIS) for spatial prediction of landslide susceptibility in Qazvin Province (Iran). To this end, the landslide inventory map, composed of 199 identified landslides, is divided into training and testing landslides with a 70:30 ratio. To create the spatial database, thirteen landslide conditioning factors are considered within the geographic information system (GIS). Notably, the spatial interaction between the landslides and mentioned conditioning factors is analyzed by means of frequency ratio (FR) theory. After the optimization process, it was shown that the DE-based model reaches the best response more quickly than other ensembles. The landslide susceptibility maps were developed, and the accuracy of the models was evaluated by a ranking system, based on the calculated area under the receiving operating characteristic curve (AUROC), mean absolute error, and mean square error (MSE) accuracy indices. According to the results, the GA-ANFIS with a total ranking score (TRS) = 24 presented the most accurate prediction, followed by PSO-ANFIS (TRS = 17), DE-ANFIS (TRS = 13), and ACO-ANFIS (TRS = 6). Due to the excellent results of this research, the developed landslide susceptibility maps can be applied for future planning and decision making of the related area.

## 1. Introduction

Slope failures are ubiquitous major disasters causing many financial and physical damages worldwide every year. Varnes and Radbruch-Hall [1] presented a definition of a landslide as any downward mass movement caused by gravity on slopes (e.g., artificial deposits, soil, and natural cliffs). Global reports state that developing countries have witnessed the majority (more than 90%) of the landslide events that have occurred around the world. Additionally, landslides are responsible for more than 17% of the reported fatalities [2]. Due to the large number of landslides that have occurred in recent decades, Iran is recognized as a landslide-prone area. It is noteworthy that the largest debris flow in the world, the Seimareh landslide, occurred in western Iran [3]. In another event, slope cutting and removal of the toe buttress triggered the Manjil landslide in 2013. It occurred on the Qazvin–Rasht freeway and led to blockage of the freeway [4]. 

As is known, landslide susceptibility mapping is an essential prerequisite for landslide risk management [5,6]. A proper landslide risk assessment entails determining the effective landslide parameters for discovering the spatial relationship between them and occurred landslides. Up to now, plenty of theories have been utilized for this purpose in many specific areas. Some of them are based on statistical rules, which aim to determine the importance of each independent landslide factor through assigning weights. Novel intelligent models are also proposed for approximating the susceptibility of an area through learning the mathematical relationship between a landslide and its related factors [7,8].

Many scholars have employed statistical-based methods for landslide susceptibility assessment [9,10,11]. Fayez, et al. [12] successfully implemented the FR model for landslide susceptibility assessment in India. In a comparative study, Chen, et al. [13] used frequency ratio (FR), weights-of-evidence (WoE), and statistical index (SI) methods for producing the landslide susceptibility map of Qianyang County (China). Considering 13 landslide-related factors, they achieved the proposed maps with 78.53%, 79.35%, and 79.40% prediction accuracies, respectively for WoE, SI, and FR models. Analytical hierarchy process (AHP) is another popular method employed in landslide susceptibility mapping [14,15]. Yan, et al. [16] used the integration of AHP with normalized FR with the cloud model for landslide susceptibility assessment. Moreover, Yang, et al. [17] investigated the landslide susceptibility modelling in Sichuan Province (China) using a spatial logistic regression (SLR) approach. They also developed a GeoDetector-based method for the proper selection of the landslide-conditioning parameters. Their findings showed that the estimation precision of the proposed model was about 11.9% higher than typical logistic regression (LR). Liu and Duan [18] conducted a comparison among the WoE, SI, and Index of Entropy (IoE) for quantitative assessment of landslide susceptibility in Shangnan County (China). According to the results, the WoE (with around 76 and 75% accuracy rate for the training and testing data, respectively) outperformed two other models. A new integrated statistical method, called B-GeoSVC, was proposed by Yang, et al. [19] as a reliable evaluative approach for both regional and local scales.

Moreover, various intelligent predictive models like the artificial neural network (ANN), adaptive neuro-fuzzy inference system (ANFIS), and support vector machine (SVM) have been promisingly employed for forecasting the landslide susceptibility risk [20,21,22]. Aditian, et al. [23] showed the superiority of the ANN (success rate = 0.734) for landslide susceptibility evaluation in Indonesia, in comparison with FR (success rate = 0.688), and LR (success rate = 0.687). Polykretis, et al. [24] examined the capability of different models of ANFIS. Based on the calculated prediction accuracies (i.e., between 0.7 to 0.90), all implemented models are reliable enough to be used for the mentioned purpose. Likewise, Chen, et al. [25] evaluated the capability of three state-of-the-art predictive models of ANFIS combined with FR (ANFIS-FR), generalized additive model (GAM), and SVM in landslide susceptibility assessment in Hanyuan County (China). This study showed that SVM presents the most accurate prediction (accuracy = 87.5%), followed by ANFIS-Fr (accuracy = 85.1%) and GAM (accuracy = 84.6%). Pham, et al. [26] proposed a hybrid predictive model named rotation forest-based radial basis function neural network for landslide susceptibility zonation of the Himalayan area, India. They found that the proposed model could be a good alternative for this aim, due to the better performance than LR, multi-layer perceptron neural network, the hybrid of rotation forest and decision trees (RFDT), and naïve Bayes (NB). 

Furthermore, many studies have focused on the development of hybrid metaheuristic algorithms incorporated with typical models in order to achieve more powerful predictive tools [27,28,29]. Nguyen, et al. [30] used particle swarm optimization (PSO) and artificial bee colony (ABC) metaheuristic techniques to optimize the performance of the ANN for landslide susceptibility mapping at northern Iran. The calculated area under the curve (AUC) values revealed that the prediction accuracy of the MLP increased from nearly 77% to around 86% and 80%, respectively by applying the PSO and ABC algorithms. Likewise, Chen, et al. [31] coupled three optimization techniques of PSO, genetic algorithm (GA), and differential evolution (DE) with the ANFIS for spatial hazard assessment of landslide in Hanyuan County (China). The AUC of all three ensembles obtained higher than 0.75. In addition, the ANFIS-DE (AUC = 0.844) emerged as the most promising ensemble technique, followed by ANFIS-GA (AUC = 0.821), and ANFIS-PSO (AUC = 0.780). Tien Bui, et al. [32] combined imperialist competitive algorithm (ICA) and relevance vector machine (RVM) for landslide susceptibility modelling of Lang Son City (Vietnam). At the same time, they considered the SVM and LR as benchmark models. They showed that the suggested RVM-ICA outperformed SVM and LR with respective AUCs of 0.92, 0.91, and 0.87, respectively. 

As mentioned above, various studies have successfully used the ANFIS for landslide susceptibility assessment [33,34]. However, hybrid ensembles of this model have been broadly used for similar applications like flood [35] and forest fire susceptibility [36] assessment. To the best knowledge of the authors, optimizing this model with metaheuristic algorithms for landslide susceptibility mapping has been rarely explored [37]. Hence, the essential novelty of this research lies in synthesizing four wise evolutionary algorithms, namely GA, PSO, DE, and ant colony optimization (ACO) with ANFIS to remedy its computational shortcomings like local minimum [38] and dimension dangers [31] in spatial modelling of the landslide. Besides, the study area (i.e., Qazvin County, Iran) is a relatively landslide-prone area that has not been sufficiently investigated in prior studies. In this regard, followed by providing the required spatial database, the FR index is calculated to measure the importance of each sub-class of the considered conditioning factors. Then, the landslide susceptibility maps are generated by each model, and the results are validated by the area under the receiving operating characteristic curve (AUROC), mean square error (MSE), and mean absolute error (MAE) accuracy criteria. The proposed models, however, may be applicable to other areas with similar environmental conditions.

## 2. Materials and Methods

### 2.1. Study Area

**Figure 1:**
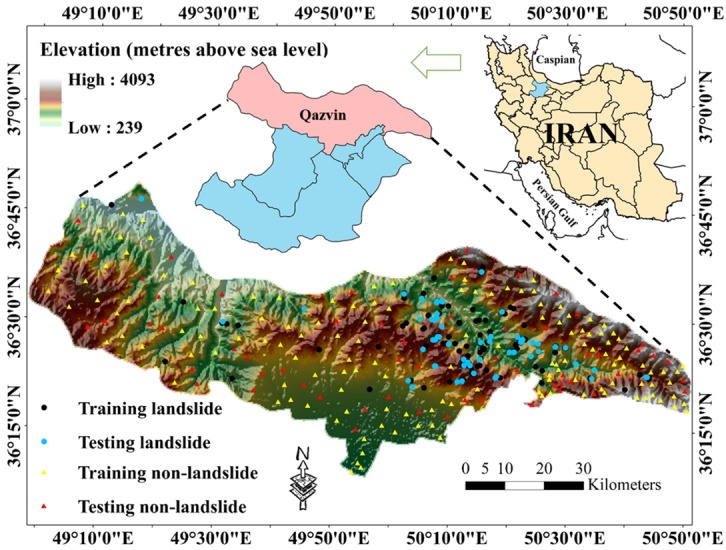
Location of the study area, and spatial distribution of landslides and non-landslide points.

The study area is Qazvin County, located in Qazvin Province, one of the 31 provinces of Iran, in the north-western part of the country. Figure 1 illustrates the exact location of Qazvin County. It covers roughly 4992 km^2^ and lies within the longitude 48°58′ to 50°51′ E and latitude 36°08′ to 36°48′ N. In the northern watersheds of Qazvin, the Shahrood river flows, which is the result of joining Taleghan and Alamut rivers [39]. Due to the presence of the Alborz mountains, this area is known to have a mountainous climate [40], and approximately half of the area is covered by mountainous pastures. The altitude ranges from 239 to 4093 m above the sea level, and it is higher than 1200 m in the major part of the area [4]. The average temperature of the hot and cold seasons is reported as approximately 28 °C and 1 °C, respectively. Also, the annual precipitation in most parts of the county is higher than 500.5 mm [40]. The slope ranges from 0° to 45°, where more than 70% of the area contains gentle slopes (i.e., slope lower than 15°). According to the Geology Survey of Iran (GSI), Qazvin County lies on a bed with 25 geology units. Among them, two groups of Dacitic andesitic volcanic tuff and sandy limestone, Marl, calcareous sandstone, and minor conglomerate are the most common rocks, covering 19.45% and 16.20% of the county, respectively. Also, the soil map shows that approximately 60% of the study area is categorized as Rock Outcrops/Entisols soil. 

The spatial distribution of the identified landslide, as well as the non-landslide points, is illustrated in Figure 1 over the hill shade map of the study area. According to this figure, the majority of slope failures have occurred in the right half of the Qazvin County, mostly along the territorial roads and detected faults.

### 2.2. Data Preparation and Spatial Relation Between the Landslide and Related Factors

**Figure 2:**
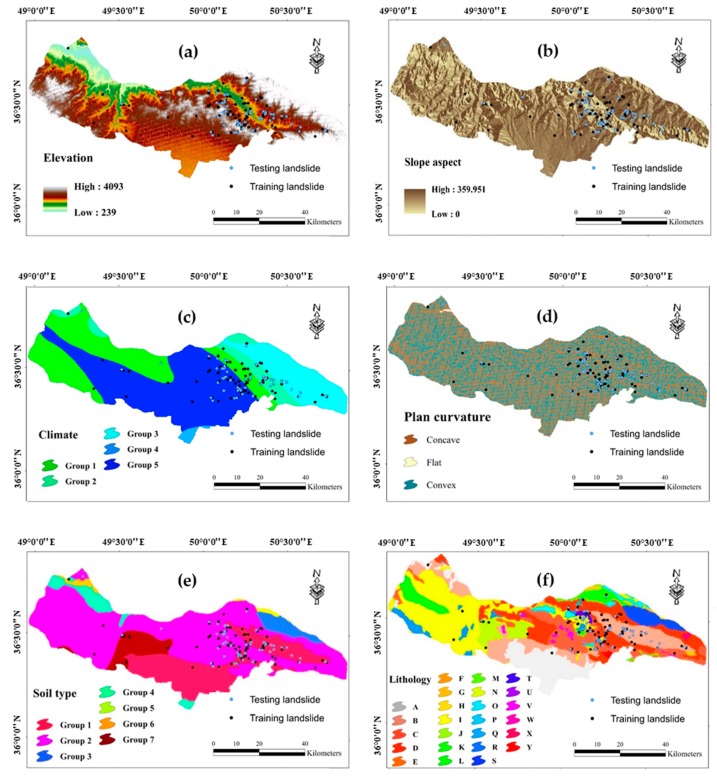

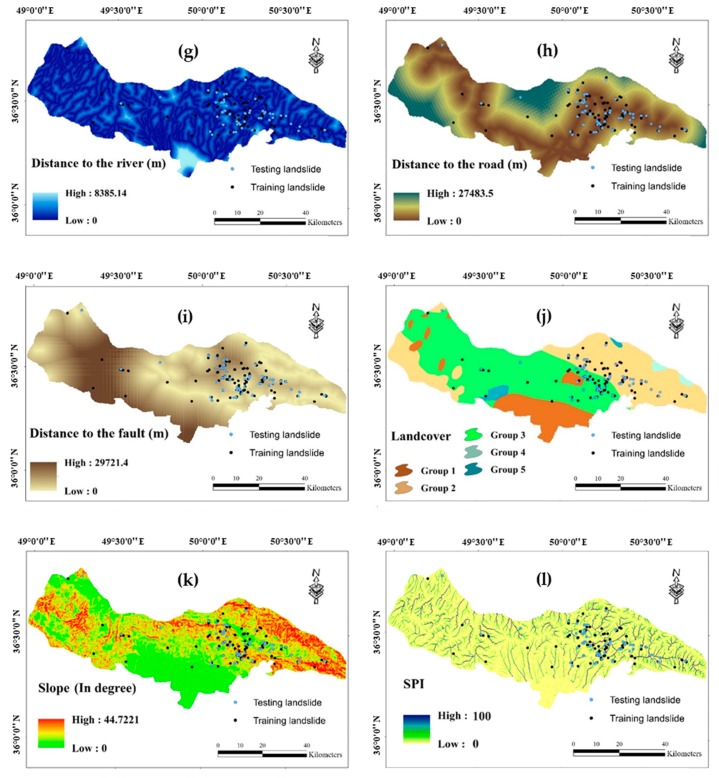

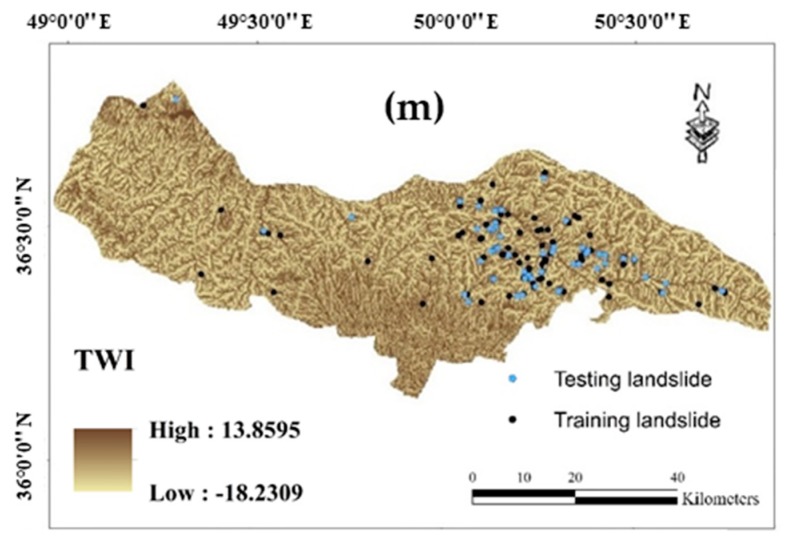
Landslide conditioning factor, percentage of the area for each sub-class, and the calculated FR for: (**a**) elevation, (**b**) slope aspect, (**c**) climate, (**d**) plan curvature, (**e**) soil type, (**f**) lithology, (**g**) distance to river, (**h**) distance to road, (**i**) distance to fault, (**j**) land cover, (**k**) slope degree, (**l**) SPI, and (**m**) TWI.

When it comes to implementing intelligent models, using a valid dataset is very important [41]. The dataset used in this study consists of thirteen landslide condition factors, including elevation, slope aspect, climate, plan curvature, soil type, lithology, distance to the river, distance to the road, distance to the fault, land cover, slope degree, stream power index (SPI), and topographic wetness index (TWI) as the input variables, and landslide occurrence (0 = no landslide and 1 = landslide) as the target variable. All layers are produced and processed in the geographic information system (GIS) with a pixel size of 10 × 10 m [42,43,44]. Figure 2 illustrates a map of the mentioned landslide-related factors.

Providing a valid landslide inventory map is an essential step in the susceptibility analysis of this natural hazard [45]. In the study area, a total of 199 landslides were marked using previous records from the national geoscience database of Iran (NGDIR), as well as satellite imagery (IRS: LISS-III) and interpreting the aerial photos (in 1:25000 scale) covering the past 20 years (i.e., 1995–2015) [46]. It should be noted that the identified landslides are mostly translational and rotational slope failures are rarely observed. Some field photographs of landslides that occurred in Qazvin Province can be found in a research by Arjmandzadeh, et al. [47]. Out of the marked landslides, 139 events (i.e., 70% of the whole dataset) are randomly selected and used for the training phase, and the remaining 60 events (i.e., 30% of the whole dataset) are allocated to the validation phase. Besides, 199 non-landslide points are randomly produced over the areas devoid of landslides and divided into the training and testing parts with the same proportions.

**Figure 3:**
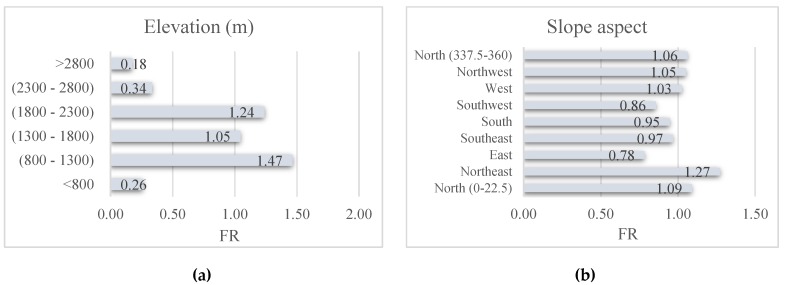

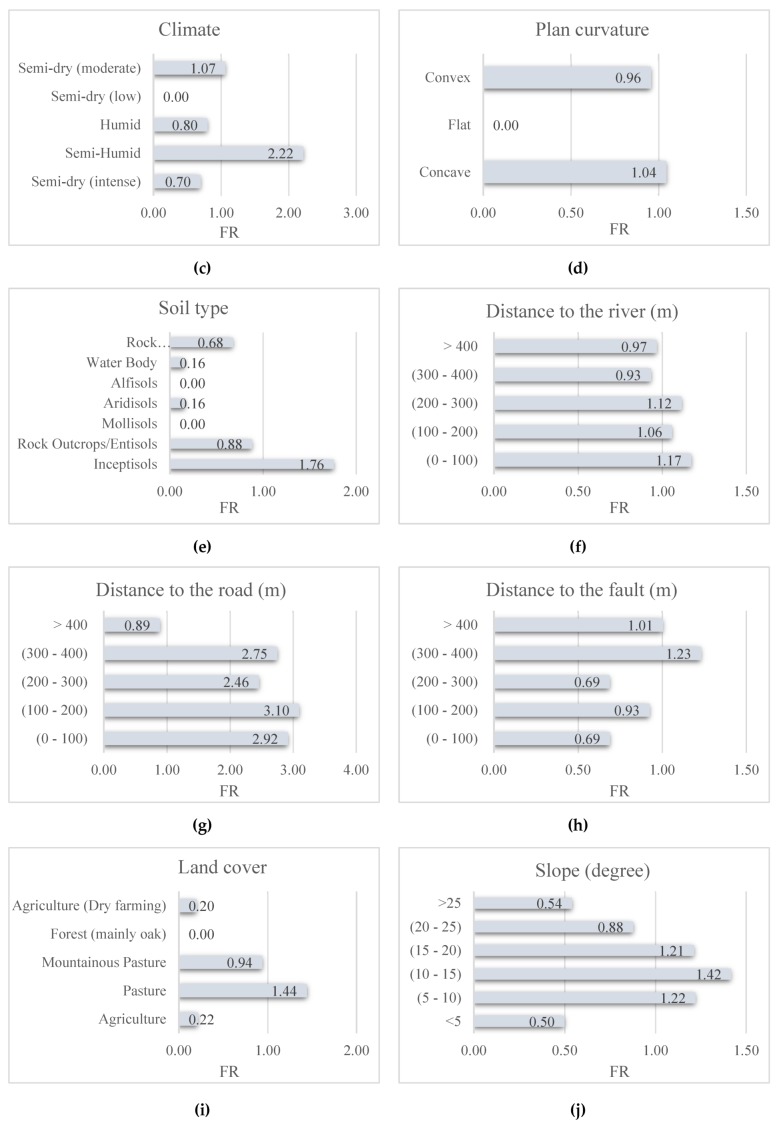

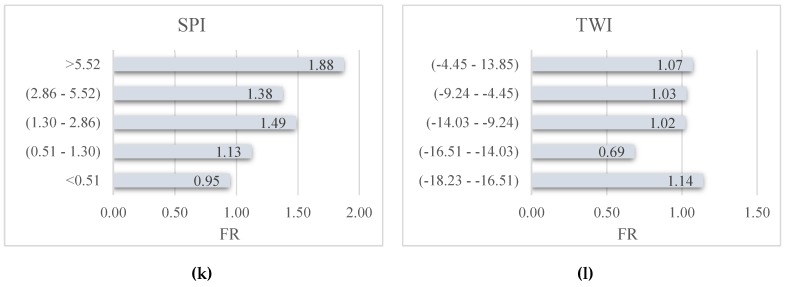
The obtained FRs for landslide conditioning factors: (**a**) elevation, (**b**) slope aspect, (**c**) climate, (**d**) plan curvature, (**e**) soil type, (**f**) distance to river, (**g**) distance to road, (**h**) distance to fault, (**i**) land cover, (**j**) slope degree, (**k**) SPI, and (**l**) TWI.

The FR theory is also considered to measure the spatial correlation between the landslides and conditioning factors. Each sub-class of the landslide conditioning layers receives an FR value. In this sense, the higher the values of FR, the more significant of the proposed sub-class [48]. This index is expressed as follows:

(1) $$FR=\frac{N_{landslide}}{N_{domain}}$$

where *N_landslide_* and *N_domain_* respectively denote the percentage of the landslide events located in the proposed sub-class and the percentage of the domain covered by it. Figure 3 shows the results of the FR analysis.

A digital elevation model (DEM) of the Qazvin County with 10 m spatial resolution was created from topographic data (contours and survey base points) at 1:25000 scale. This layer is subsequently used for generating the GIS layer of some landslide conditioning factors including elevation, plan curvature, slope aspect, and slope degree of the study area. The altitude varies in the range of 239−4093 m, and more than 70% of the area is above 700 m. The elevation layer was classified into six classes with 500 m intervals [49] and the highest FR is obtained for the elevations between 800 and 1300. Based on the values in the range of [0–360°], the slope aspect layer is classified into nine groups including North (0–22.5°), North-East (22.5–67.5°), East (67.5–112.5°), South-East (112.5–157.5°), South (157.5–202.5°), South-West (202.5–247.5°), West (247.5–292.5°), North-West (292.5–337.5°) and North (337.5–360°). The FR analysis shows that North (0–22.5°), North-East, North-West, West, and North (337.5–360°) have this value larger than 1. Plan curvature was classified into three categories of Concave (<−0.001), Flat (−0.001–0.001), and Convex (>0.001) [50], which the first group is more important due to the calculated FR = 1.04. The slope of the study area ranges from 0 to 45°, where the majority of them are gentle slopes (i.e., lower than 15°). The slope layer was classified into six groups with 5° intervals [51,52]. The obtained FR for three groups of (5–10°), (10–15°), and (15–20°) are 1.22, 1.41, and 1.21, respectively, which shows more correlation of these groups in comparison with (0–5°), (20–25°), and >25°.

Five different climates are found for the proposed area labeled as semi-dry (intense), semi-humid, humid, semi-dry (low), and semi-dry (moderate). Around 9% of the area is under the second climate with FR = 2.21. Seven categories form the soil type map of Qazvin including Inceptisols, Rock Outcrops/Entisols, Mollisols, Aridisols, Alfisols, Water Body, and Rock Outcrops/Inceptisols. Among these, the first category emerges as the most significant one for landslide occurrence. Based on the lithology map, provide from Geology Survey of Iran (GSI), twenty-five different rocks form the geology of the area. The description of these units and the calculated FR are presented in Table 1. Note that, the lithology units shown by TRJs and gb have shown the highest sensitivity to the FR analysis with respective values of 5.80 and 5.54. 

The effect of the linear phenomena including the distance from the river, road, and fault is taken into consideration by calculating the Euclidean distance [20] from them. Consequently, three GIS layers of distance to the river, distance to the road, and distance to the fault are generated and classified into five categories with 100 m intervals [53,54]. Accordingly, the most significant of them are (0–100) m (FR = 1.17), (100–200) m (FR = 3.09), and (300–400) m (1.23), respectively. Based on the land use map, five utilizations of land are found for the study area including “Agriculture”, Pasture, Mountainous Pasture, Forest (mainly oak), and Agriculture (Dry farming). Among these, the largest FR = 1.44 is obtained for the second label.

Finally, for applying the geo-morphometric impacts, two secondary factors of SPI and TWI are calculated based on Equations (2) and (3) [55,56]. This is noteworthy that these factors represent the erosion power of streams and the amount of accumulated water in a place, respectively.

(2) SPI= α × tanβ,

(3) TWI=ln (α/tanβ),

in which *α* and *β* are the specific catchment and gradient, respectively.

### 2.3. Methodology

**Figure 4:**
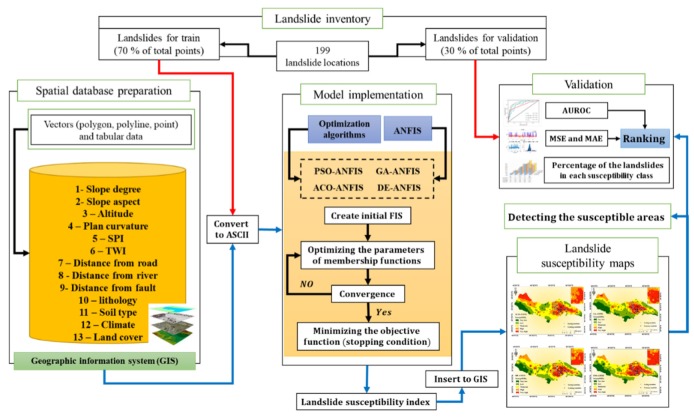
The methodology of the applied procedure for landslide susceptibility assessment.

Figure 4 shows an overall view of the steps were taken to achieve the goal of the study. Briefly, after providing a proper spatial database, the existing GIS rasters were converted into ASCII format. The proposed GA, PSO, DE, and ACO metaheuristic algorithms were designed and coupled with ANFIS in the programming language of MATLAB 2014. Each model performed to estimate the landslide susceptibility index. Then, the landslide susceptibility map of each model is produced in the GIS environment, using the produced values. Finally, three accuracy criteria including the AUROC, MSE, and MAE are defined to evaluate the efficiency of the implemented techniques. A score-based ranking system is also developed to compare the efficacy of the models. Equations (4) and (5) express the formulation of the MSE and MAE error criteria:

(4) $$MSE=\frac{1}{N}\sum_{i=1}^{N}{\left(Y_{i_{observed}\;}-Y_{i_{predicted}\;}\right)}^{2},$$

(5) $$MAE=\frac{1}{N}\sum_{i=1}^{N}\left(Y_{i_{observed}\;}-Y_{i_{predicted}\;}\right),$$

in which *N* shows the number of involved instances, and *Y_i_observed__* and *Y_i_predicted__* stand for the desired and estimated values of landslide susceptibility index, respectively.

In the following section, a description of the ANFIS, GA, PSO, DE, and ACO models is presented. 

#### 2.3.1. Adaptive Neuro-Fuzzy Inference System

The name adaptive neuro-fuzzy inference system (ANFIS) [57] denotes a combination of ANN and fuzzy-based system to capture the benefits of both of them. In other words, the ANN is employed to optimize the implemented fuzzy theory for more flexibility of approximation [58]. Utilizing if-then rules [59] enables ANFIS to present a reliable prediction from various complex problems. It synthesizes two learning methods of back-propagation gradient descent and least-squares for discerning the mathematical relationship between sets of input-output data. The performance of the ANFIS can be expressed in five steps: In the first layer, the membership function (MF) values of the input variables are calculated. Next, the rule firing strength is calculated and normalized in the second and third layers, respectively. The outputs of the consequent part are produced in Layer 4, and eventually, the ANFIS releases the final output from fifth layer.

#### 2.3.2. Genetic Algorithm

Genetic algorithm (GA) is heuristic search techniques first suggested by Holland [60]. This algorithm follows Darwin’s natural selection principles for finding the optimal solution to a defined problem. Darwin’s survival evolution theory states that the living organism in the future generation is more suitable than the former one. Generally, five major operators of GA are random number creator, fitness assessment unit, the genetic operator (i.e., for reproduction), crossover operator, and mutation operator. A mathematically-defined problem, containing some computational parameters that need to be optimized, is the input of the GA algorithm. Firstly, a so-called input vector chromosome is selected. A set of these vectors constructs the colony of GA. Based on biological evolution, the developed created colony grows and evolves under certain conditions. A function is defined for each chromosome to evaluate its fitness. Then, the more robustness of the chromosome, the more likelihood of survive and reproduce. The new generation (i.e., children) is produced through the crossover and mutation operations. In fact, a child is a result of combining the content of two existing chromosomes. During a mutation process, a child may receive a new gene which is not from the side of his parents. The same process is carried out for the new generation to achieve the optimal solution [61]. Note that, the algorithm continues performing until one of the goals (e.g., the error or maximum iterations) is met.

#### 2.3.3. Particle Swarm Optimization

The main idea of particle swarm optimization (PSO) method is extracted from the social behavior of bird flocking or fish schooling in the real world. This stochastic method was first suggested by Kennedy and Eberhart [62]. The PSO initialized with a set of random solutions and updates the generation for achieving the optimal situation of the problem. Possible solutions, called particles, fly through the problem space. In this movement, they follow their optimum colleagues. Similar to other optimization techniques, the goodness of each particle is evaluated by a fitness function. Each particle saves the track of its coordinates which gives the best fitness value under the name p_best_. Another so-called elite position g_best_ is defined indicating the best position obtained from all of the particles so far. The PSO aims to change the velocity (*R_i_*) of each particle toward the location of the discovered p_best_ and g_best_. Assuming *N_i_* as the particle position, this process can be expressed as follows [63,64]:

(6) $$N_{i}^{\prime }=w\;N_{i}+C_{1}r_{1}\;\left(p_{best}-R_{i}\right)+C_{2}r_{2}\;\left(g_{best}-R_{i}\right),$$

(7) $$R_{i}^{\prime }=R_{i}+N_{i}^{\prime },$$

in which *C_1_* and *C_2_* respectively indicate the cognitive and social scaling parameters. Also, *w* denotes the inertia weight, and *r_1_* and *r_2_* symbolize the random numbers between 0 and 1.

#### 2.3.4. Differential Evolutionary Algorithm

Differential evolutionary (DE) is a recently developed hybrid evolution algorithm, which was first presented by Storn and Price [65]. It has been widely employed to find the globally optimal solution for an issue defined in continuous space [65,66]. Similar to other optimization methods, the DE gets started by a random production process, for generating the initial population. Three major steps of mutation, crossover, and selection are considered to achieve the optimal solution. In the mutation step, each individual $X_{i}^{G}$ is employed to produce the mutant vector (i.e., donor vector) $V_{i}^{G}$. [67,68]. During the crossover step, the crossover operators produce a trial vector $U_{ji}^{G}$. To do this, a number of relations of the $X_{i}^{G}$ should be replaced with the mutant vector $V_{i}^{G}$. Lastly, at the selection step, the goodness value of the $U_{i}^{G}$ with $X_{i}^{G}$ are compared for introducing the best choice for the next generation [65].

#### 2.3.5. Ant Colony Optimization

The primary version of ant colony optimization (ACO) method was known as the ant system which was first designed for optimizing the travelling salesman problem [69]. In fact, the main effort of this work is to find the shortest path to link the intended cities [70]. In the case of intelligent models, like ANN and ANFIS, this algorithm aims to achieve the optimal computational parameters of these networks. A presentable solution is made of some components which are added by ants in each iteration. The ants consider a probability for selecting these components. Two determinant factors in this process are the pheromone and the heuristic factors which respectively reflect the past experience of the relations and the tendency for choosing a component regarding the defined objective function (OF). In the ACO method, the shortest path is discovered by the artificial ants through leaving a chemical pheromone trail along the crossed path. They do this for guiding other relations. As a result, the most promising track is distinguished. Assuming $\tau_{ij}^{\alpha }$ and $\eta_{ij}^{\beta }$ as the pheromone value and heuristic factors, respectively, Equation (8) formulates the mentioned probability:

(8) $$P_{ij}^{q}\left(t\right)=\frac{\tau_{ij}^{\alpha }\times \eta_{ij}^{\beta }}{\sum_{u\in N_{i}^{q}\;}\tau_{iu}^{\alpha }\times \eta_{iu}^{\beta }},$$

in which *N_i_* defines a neighborhood of the node *i*.

## 3. Results

This paper investigates the efficiency of four evolutionary ensembles of a fuzzy-based model, namely GA-ANFIS, PSO-ANFIS, DE-ANFIS, and ACO-ANFIS for landslide susceptibility assessment. Qazvin County in Iran is selected as the study area. In this work, considering the common ratio of 70:30, 139 landslides were specified to the training phase, and the remaining 60 landslide points were used to measure the accuracy of the applied models. In the next step, four hybrid stochastic algorithms of GA, PSO, DE, and ACO were synthesized with the ANFIS predictive model for fine-tuning the ANFIS MFs [71]. In detail, when it comes to optimization algorithms, they need some computational parameters. Regarding the used basic model, these computational parameters can vary (e.g., the weights and biases in the ANNs). In the case of ANFIS, the mentioned optimization algorithms aim to find the optimal values for the parameters of MFs. Notably, ANFIS is known as a capable model due to having a combination of fuzzy inference system (FIS) expert knowledge and neural learning ability [34]. It has been promisingly used for landslide hazard analysis in previous researches [58,72]. At the beginning of the ANFIS performance, it constructs a basic FIS. The computational units are then extracted and updated by evolutionary algorithms. Eventually, they are applied to develop the optimized model.

**Figure 5:**
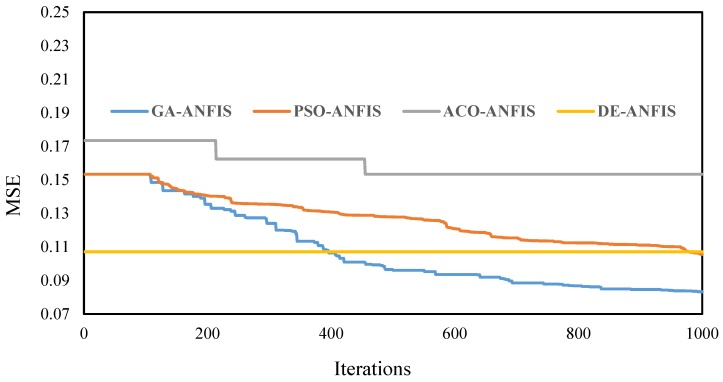
The convergence curves of the cost functions for the used models.

The number of repetitions for all models determined as 1000 to give enough opportunity for decreasing the error. Meanwhile, the MSE between the actual and predicted landslide susceptibility indices were defined as the cost function. Figure 5 depicts the convergence diagram of the GA-ANFIS, PSO-ANFIS, DE-ANFIS, and ACO-ANFIS. As is seen, the algorithms have shown different behaviors for optimizing the ANFIS. The GA-ANFIS, as well as PSO-ANFIS, have started decreasing MSE after 100th iteration. The corresponding curves are on a continuously downward path. The GA-based ensemble surpasses the PSO-ANFIS and reaches a lower MSE in the final (0.08333771 vs. 0.105558762). Unlike these, the DE-ANFIS did not show any sensitivity to the number of iterations and remained steady till the end. The MSE for this model obtained 0.107146933 which shows a very close value to the cost function of the PSO-ANFIS. As for the ACO-ANFIS, it started with a relatively-high MSE and reduced it to 0.153427807 within three steps. All in all, DE-ANFIS can be introduced as the fastest ensemble.

**Figure 6:**
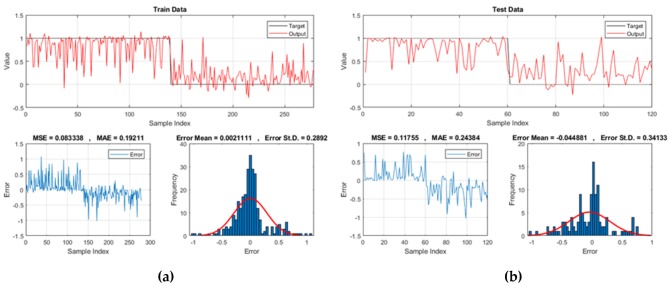

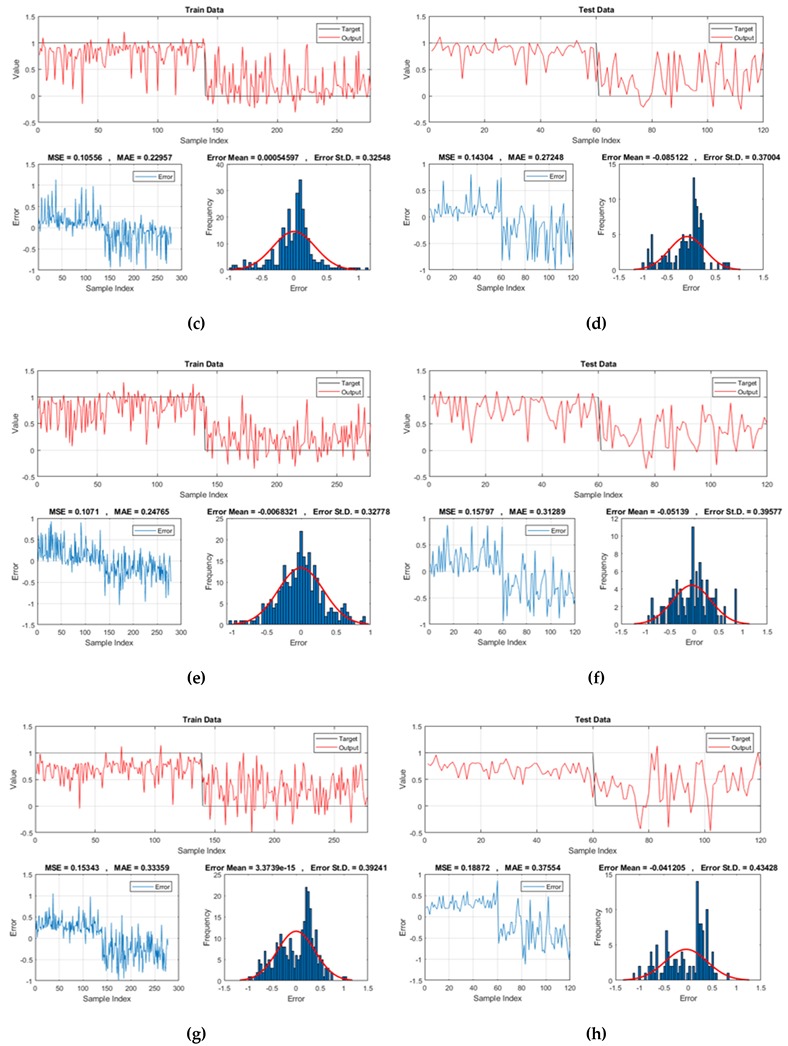
The results obtained for (**a**) and (**b**) GA-ANFIS, (**c**) and (**d**) PSO-ANFIS, (**e**) and (**f**) DE-ANFIS, (**g**) and (**h**) ACO-ANFIS, respectively for the training and testing samples.

The results of the training and testing performance of the models are presented in Figure 6 in three parts: (i) A graphical comparison between the actual (targets) and estimated (response) landslide susceptibility indices, (ii) A schematic view of the calculated error (i.e., the difference between the targets and system responses), and (iii) A histogram chart showing the frequency of each error value. To create the landslide susceptibility maps of the implemented models, the produced landslide susceptibility indices were extracted and inserted to ArcGIS. Additionally, Natural break classification method [73,74] was applied to classify the resulted maps into five susceptibility categories including Very-low, Low, Moderate, High, and Very-high susceptible.

**Figure 7:**
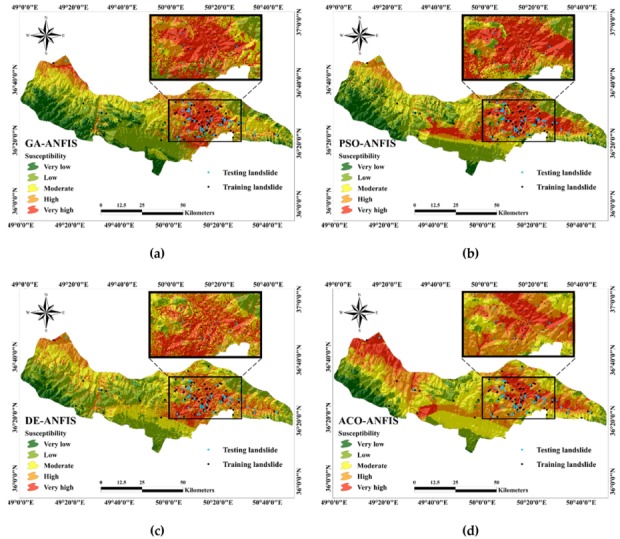
Generated landslide susceptibility maps for (**a**) GA-ANFIS, (**b**) PSO-ANFIS, (**c**) DE-ANFIS, (**d**) ACO-ANFIS.

Figure 7 shows the generated maps of the GA-ANFIS, PSO-ANFIS, DE-ANFIS, and ACO-ANFIS predictions.

As is seen, all four applied ensembles have rightly specified a high level of susceptibility to the areas with a large aggregation of the landslide points (see the marked area in Figure 7). In addition, they have fittingly classified the areas devoid of the landslide (mostly in the South and South-West of the studied area) as low and very low susceptible.

The percentage of each susceptibility map is also calculated. Accordingly, around 28% (1418 km^2^), 33% (1639 km^2^), 31% (1568 km^2^), and 46% (2315 km^2^) of the studied area is recognized to be under the high landslide occurrence risk (i.e., high and very high susceptibility classes), respectively from the side of GA-ANFIS, PSO-ANFIS, DE-ANFIS, and ACO-ANFIS ensembles. Besides, the largest percentage of the safe areas (very low and low categories) are obtained for the GA-ANFIS (15.48% and 30.82% respectively). The percentage of the training and testing landslide points located in each susceptibility class are also calculated and presented in Table 2. According to this table, 78.57%, 76.19%, 81.75%, and 88.10% of the training landslides, as well as 80.00%, 80.00%, 81.67%, and 93.33% of the testing landslides are rightly located in the landslide-prone areas by GA-ANFIS, PSO-ANFIS, DE-ANFIS, and ACO-ANFIS, respectively. Considerably, these values are obtained < 3% in the areas labeled as very low susceptibility.

**Figure 8:**
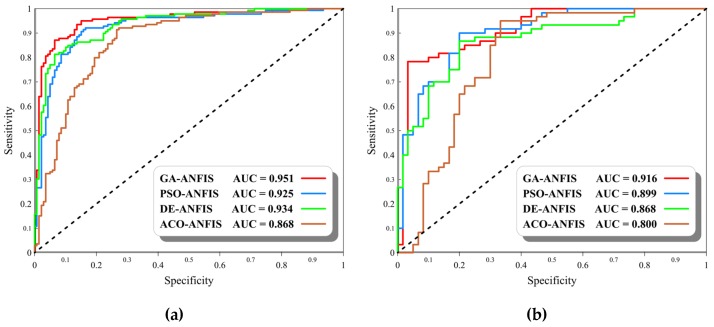
Obtained for the (**a**) training data and (**b**) testing data.

For evaluating the accuracy of the generated landslide susceptibility maps, the ROC curve was plotted. As is known, the area under the ROC curve is a good accuracy indicator for diagnostic issues [75]. These value ranges from 0.5 to 1 so that the quality of the prediction is directly proportional to the AUROC value. The ROC curves of the implemented models are shown in Figure 8a,b, respectively, for the training and testing landslides.

The obtained values of all three accuracy criteria (i.e., MSE, MAE, and AUROC) are summarized in Table 3. A score-based ranking system is also developed within this table for better distinguish of the most capable model. Each accuracy criterion is considered for both training and testing phases to receive a ranking score varying between 1 to 4. In this regard, the more accuracy the index represents, the higher score is assigned to it. As is seen, in the training phase, the obtained MSEs and MAEs for GA-ANFIS (0.0833 and 0.1921), PSO-ANFIS (0.1055 and 0.2295), DE-ANFIS (0.1071 and 0.2476), and ACO-ANFIS (0.1534 and 0.3335) indicate a lower prediction error for the GA- and PSO-based ensembles. After those, the DE-based model outperformed the ACO-ANFIS. This claim can also be supported by respective calculated AUROCs 0.951, 0.925, 0.934, and 0.868. However, the AUROC of the DE-ANFIS is slightly higher than PSO-ANFIS.

The testing results show a good generalization power of all used models. Accordingly, the obtained MSEs and MAEs for GA-ANFIS (0.1175 and 0.2438), PSO-ANFIS (0.1430 and 0.2724), DE-ANFIS (0.1579 and 0.3128), and ACO-ANFIS (0.1887 and 0.3755) reveal that the first model has predicted the landslide hazard index more efficiently than other three models. Also, the PSO-ANFIS emerged as the second accurate model, followed by DE-ANFIS and ACO-ANFIS. Besides, the values of the AUROC represent 91.6%, 89.9%, 86.8%, and 80.0%. Prediction capability for the GA-ANFIS, PSO-ANFIS, DE-ANFIS, and ACO-ANFIS, respectively. 

All in all, considering a total ranking score (TRS) as the summation of the scores obtained based on the mentioned indices, the GA-ANFIS (TRS = 24), emerged as the most promising model for spatial prediction of landslide hazard in the Qazvin county. After that, the PSO-ANFIS (TRS = 17), DE-ANFIS (TRS = 13), and ACO-ANFIS (TRS = 6) presented an acceptable prediction accuracy.

## 4. Discussion

Due to the devastating impacts of the landslides, susceptibility assessment of this natural hazard has received increasing attention during the last decades [76]. This study addresses the applicability evaluation of four hybrid integration techniques using the adaptive neuro-fuzzy inference system coupled with a genetic algorithm, particle swarm optimization, differential evolution and ant colony optimization for landslide susceptibility analysis in Qazvin Province (Iran). The prediction accuracy of the designed models was evaluated and compared using the ROC diagram and the AUROC index. As an advantage of the ANFIS, the basis of this predictive tool is a combination of neural learning of the ANNs and expert knowledge of FIS [34]. Some researchers have promisingly employed this model for landslide susceptibility mapping [34,77,78]. Oh and Pradhan [79] revealed that the ANFIS acts very effectively for regional assessment of landslide susceptibility. They tested various MFs embedded in this model for estimating the landslide susceptibility values in a prone district of Penang Island (Malaysia). The findings of the mentioned research demonstrated that the accuracy of the landslide susceptibility maps obtained from trapezoidal, triangular, polynomial and generalized bell MFs (accuracy ≈ 84%) surpass the ones produced by four different Gaussian and sigmoidal MFs.

As stated above, the ANFIS has emerged as one of the most powerful machine learning approaches, but some drawbacks associated with this tool (e.g., the non-adjutancy of membership) drive us to use hybrid evolutionary algorithms for optimizing its performance. In this paper, four wise optimization techniques of GA, PSO, DE, and ACO were applied to find the optimal values of the parameters of the ANFIS. More clearly, these algorithms are stochastic search schemes which perform in a repetitive loop to minimize a defined objective function. This process results in finding the most appropriate solution amongst a huge number of candidates.

The results indicated that all used models perform with good accuracy for the mentioned purpose. Having a look at the obtained accuracy criteria, as well as the spatial interaction between the landslide points and the susceptibility classes, it can be concluded that the GA-ANFIS (TRS = 24) excelled other designed ensembles. The PSO-ANFIS (TRS = 17) appeared to be the second accurate model, followed by the DE-ANFIS (TRS = 13) and ACO-ANFIS (TRS = 6). However, in similar research by Chen, Panahi and Pourghasemi [31], the superiority of the DE-based ensemble was stated (i.e., in comparison with PSO- and GA-based models), the outcomes of the present study confirm that the GA outperforms PSO for optimizing the parameters of the proposed fuzzy system. In fact, the discrete optimizing method of the GA surpassed the constant method of the PSO. More specifically, the latter algorithm allocates memory for maintaining every promising solution of all particles. This is while, prior knowledge of the GA population is antiquated as the new population is involved. Notably, the results of our study show more robustness in comparison with the mentioned reference. 

Reaching the lowest training error (Figure 5) is, perhaps, the main reason for the excellent generalization power of the GA-ANFIS (91.6% accuracy). We say perhaps because despite a slightly smaller OF obtained for the PSO-ANFIS (0.1055) compared to DE-ANFIS (0.1071), it gave less training accuracy (92.5% vs. 93.4%). Moreover, not surprisingly, the appreciable distinction between the learning capability of the ACO-AFIS and other models (i.e., larger OF) led to weaker performance for estimating the landslide susceptibility values of the testing points. Altogether, there was no disagreement between the capability of learning a pattern and generalization power for all models used in this study. In other words, each model that excelled in the training phase, was superior in the testing phase, too. 

Setting the performance of the applied algorithms aside, the number of iterations that each model needed to reach the lowest OF was considered as the main factor for evaluating the convergence speed of that model (i.e., within 1000 repetitions and regardless the time). It was deduced that the DE-ANFIS does not show any sensitivity to the number of repetitions, and the first try is the best one. It should be noted about DE that, unlike other metaheuristic techniques, the vectors of the current generation in this technique are created through random sampling and combining the vectors belonging to the former generation. Also, the real-valued mutation and crossover factors cause the convergence of the search action [80]. Similar to the ACO-ANFIS which Termeh, et al. [81] used for flood susceptibility modelling, it minimized the OF after a few major fluctuations. As for the PSO- and GA-based ensembles, both models continued decreasing the error until the end. Now, the main question is “which ensemble can be introduced as the most suitable model, considering both accuracy and time-effectiveness”? For answering this question, it should be mentioned that there is a nuance (0.0238 of MSE, 0.0555 of MAE, and 0.017 of AUROC) between the learning quality of the controversial models of this study (i.e., GA-ANFIS and DE-ANFIS). Hence, when the time comes out as a determinant factor, the DE-ANFIS is a more appropriate ensemble for landslide susceptibility assessment. In contrast, in cases that accuracy plays a more important role, utilizing the GA-ANFIS seems more reasonable.

The methodology proposed in this paper had some limitations too. Using the original input configuration (i.e., thirteen landslide conditioning factors) resulted in the generation of high-dimensionality networks which negatively influence the complexity and the computation time of the models. This issue can be solved by optimizing the input factors by metaheuristic schemes [28]. Also, taking the effect of other environmental parameters (e.g., local drainage networks) into account could be of interest for future studies. Moreover, referring to the convergence behavior of the elite models (i.e., the GA and PSO) in Figure 5, they are able to achieve more accurate understanding from the landslide pattern for further iterations. This is while this parameter was bound to 1000 in this study.

## 5. Conclusions

The importance of landslide susceptibility mapping for alleviating the damages triggered by this natural hazard is obvious. Due to the complexity and non-linearity of such modellings, the application of new intelligent tools like ANN and ANFIS has antiquated many traditional methods. Despite various benefits of these models, some difficulties like dimension dangers and local minimum still exist. Such problems have driven scholars to employ hybrid optimization algorithms for optimizing the performance of typical models, especially for high-dimensional problems like landslide susceptibility modelling. In the present study, firstly, the FR theory was used to assess the spatial interaction between the landslides and considered conditioning factors. Then, four wise metaheuristic algorithms, namely GA, PSO, DE, and ACO were employed to train the ANFIS, for proper landslide susceptibility analysis in Qazvin County (Iran). In fact, the main duty of the mentioned algorithms is to find the most appropriate computational parameters of the proposed fuzzy system, in order to decrease the impact of dimension dangers and local minimum. As the first outcome, the DE-ANFIS model emerged as the fastest ensemble for minimizing the OF. Eventually, the landside susceptibility maps were produced and the AUROC criterion was used to evaluate the accuracy of them. Referring to the obtained accuracies of 91.6%, 89.9%, 86.8%, and 80.0%, respectively for the GA-ANN, PSO-ANFIS, DE-ANFIS, and ACO-ANFIS ensembles, it was concluded that the GA outperforms other implemented algorithms in optimizing the performance of the ANFIS. The produced landslide susceptibility maps can be applied for proper decision making and risk management of landslide in the studied area. However, the results indicate that the designed models are accurate enough to be an alternative for the mentioned purpose, the authors believe that more accuracy can be achieved by applying various ideas such as simultaneous optimization of the input combination and the used predictive model, which seems a good subject for future studies.

## Figures and Tables

**Table 1:** The description of the lithology units.

Name	Symbol	Description	Geological Age	Age Era	FR
A	Qft1	Vally terrace deposits and high level piedmont fan	Quaternary	Cenozoic	0.1342
B	Mm,s,l	Calcareous sandstone, Marl, sandy limestone, and minor conglomerate	Miocene	Cenozoic	1.8086
C	Ek	Well bedded green tuff and tuffaceous shale (KARAJ FM)	Eocene	Cenozoic	1.8527
D	Ebv	Basaltic volcanic rocks	Middle. Eocene	Cenozoic	1.4191
E	Ek.a	Calcareous shale with subordinate tuff (Asara Shale)	Middle. Eocene	Cenozoic	0.0000
F	Pr	Dark grey medium-bedded to massive limestone (RUTEH LIMESTONE)	Permian	Paleozoic	0.9016
G	TRJs	Dark grey shale and sandstone (SHEMSHAK FM.)	Triassic-Jurassic	Mesozoic	5.8083
H	Eksh	Greenish-black shale and partly tuffaceous with intercalations of tuff (Lower Shale Member)	Middle. Eocene	Cenozoic	0.0000
I	Qft2	Low level piedment fan and vally terrace deposits	Quaternary	Cenozoic	0.9509
G	Edavt	Dacitic andesitic volcanic tuff	Middle-Late. Eocene	Cenozoic	0.1144
K	Pgkc	Light-red coarse grained, and polygenic conglomerate with sandstone intercalations	Paleocene-Eocene	Cenozoic	1.0196
L	Ogr-di	Granite to diorite	Oligocene	Cenozoic	0.0000
M	Eav	Andesitic volcanics	Middle. Eocene	Cenozoic	0.8304
N	Kbv	Basaltic volcanic	Early. Cretaceous	Mesozoic	0.0000
O	Ktzl	Thick bedded to massive, and white to pinkish orbitolina bearing limestone (TIZKUH FM)	Early. Cretaceous	Mesozoic	0.0000
P	TRe	Thick bedded grey o’olitic limestone, thin-platy, yellow to pinkish shaly limestone with worm tracks and well to thick-bedded dolomite and dolomitic limestone (ELIKAH FM.)	Early-Middle. Triassic	Mesozoic	0.0111
Q	gb	Gabbro	Eocene	Cenozoic	5.5427
R	Edav	Dacitic to Andesitic volcanic	Eocene	Cenozoic	0.4563
S	Cb	Limestone, alternation of dolomite, and verigated shale (BARUT FM)	Cambrian	Paleozoic	0.0000
T	Jl	Light grey, and thin-bedded to massive limestone (LAR FM)	Jurassic-Cretaceous	Mesozoic	3.5884
U	Edt	Rhyolitic to rhyodacitic tuff	Eocene	Cenozoic	2.6755
V	Qabv	Andesite to basaltic volcanics	Quaternary	Cenozoic	0.2398
W	Odi	Diorite	Oligocene	Cenozoic	0.7280
X	Ekgy	Gypsum	Late. Eocene	Cenozoic	0.0000
Y	Ebt	Basaltic tuff	Eocene	Cenozoic	0.0000

**Table 2:** The percentage of the training and testing landslides in each susceptibility classes.

Susceptibility Class	GA-ANFIS		PSO-ANFIS		DE-ANFIS		ACO-ANFIS	
	Train	Test	Train	Test	Train	Test	Train	Test
Very low	1.51	0.00	0.91	0.00	0.00	0.00	1.16	0.00
Low	4.49	4.14	2.98	0.00	2.52	2.99	1.82	1.99
Moderate	11.85	10.43	7.40	0.50	11.43	7.52	10.10	4.06
High	14.03	9.97	13.67	1.31	22.37	19.79	32.92	33.36
Very high	68.13	75.46	75.04	98.19	63.67	69.71	54.00	60.58

**Table 3:** The ranking system based on the results of the spatial prediction of landslide susceptibility.

Ensemble Models	Network Results						Ranking Score						Total Ranking Score (TRS)	Rank
	Training Phase			Testing Phase			Training Phase			Testing Phase				
	MSE	MAE	AUROC	MSE	MAE	AUROC	MSE	MAE	AUROC	MSE	MAE	AUROC		
GA-ANFIS	0.0833	0.1921	0.951	0.1175	0.2438	0.916	4	4	4	4	4	4	24	1
PSO-ANFIS	0.1055	0.2295	0.925	0.1430	0.2724	0.899	3	3	2	3	3	3	17	2
DE-ANFIS	0.1071	0.2476	0.934	0.1579	0.3128	0.868	2	2	3	2	2	2	13	3
ACO-ANFIS	0.1534	0.3335	0.868	0.1887	0.3755	0.800	1	1	1	1	1	1	6	4
